# Retraction: RNA-sequencing identified miR-3681 as a negative regulator in the proliferation and migration of cervical cancer cells *via* the posttranscriptional suppression of HGFR

**DOI:** 10.1039/d1ra90052h

**Published:** 2021-01-27

**Authors:** Laura Fisher

**Affiliations:** Royal Society of Chemistry Thomas Graham House, Science Park, Milton Road Cambridge CB4 0WF UK advances-rsc@rsc.org

## Abstract

Retraction of ‘RNA-sequencing identified miR-3681 as a negative regulator in the proliferation and migration of cervical cancer cells *via* the posttranscriptional suppression of HGFR’ by Fan Shi *et al.*, *RSC Adv.*, 2019, **9**, 22376–22383, DOI: 10.1039/C9RA01785B.

The Royal Society of Chemistry hereby wholly retracts this *RSC Advances* article due to concerns with the reliability of the data. The images in the article, and the raw data provided by the authors, were screened by an image integrity specialist.

The blots and many other features of the article were found to closely resemble the blots and features of other articles, which is highly unexpected given that there are completely different author lists for these articles. The western blots in these papers are very regular-shaped and do not look genuine.

Furthermore, analysis of the western blot raw data provided by the authors for this paper showed that it did not match with any of the western blots in the article, and therefore cannot be used to validate the published data.

Given the significance of the concerns about the validity of both the data in the article and the raw data provided by the authors, the findings presented in this article are not reliable.

The authors have been informed but have not responded to any correspondence regarding the retraction.

Signed: Laura Fisher, Executive Editor, *RSC Advances*

Date: 15^th^ January 2021

## Supplementary Material

